# Higher-Order Pattern Anti-Unification in Linear Time

**DOI:** 10.1007/s10817-016-9383-3

**Published:** 2016-07-27

**Authors:** Alexander Baumgartner, Temur Kutsia, Jordi Levy, Mateu Villaret

**Affiliations:** 1 Research Institute for Symbolic Computation (RISC), Johannes Kepler University, Linz, Austria; 2 Artificial Intelligence Research Institute (IIIA), Spanish Council for Scientific Research (CSIC), Barcelona, Spain; 3 Departament d’Informàtica i Matemàtica Aplicada (IMA), Universitat de Girona (UdG), Girona, Spain

**Keywords:** Generalizations of lambda terms, Anti-unification, Higher-order patterns

## Abstract

We present a rule-based Huet’s style anti-unification algorithm for simply typed lambda-terms, which computes a least general higher-order pattern generalization. For a pair of arbitrary terms of the same type, such a generalization always exists and is unique modulo $\alpha$-equivalence and variable renaming. With a minor modification, the algorithm works for untyped lambda-terms as well. The time complexity of both algorithms is linear.

## Introduction

The anti-unification problem of two terms $t_1$ and $t_2$ is concerned with finding their generalization, a term *t* such that both $t_1$ and $t_2$ are instances of *t* under some substitutions. Interesting generalizations are the least general ones. The purpose of anti-unification algorithms is to compute such least general generalizations (lggs).

For higher-order terms, in general, there is no unique higher-order lgg. Therefore, special classes have been considered for which the uniqueness is guaranteed. One of such classes is formed by higher-order patterns. These are $\lambda$-terms where the arguments of free variables are distinct bound variables. They were introduced by Miller [28] and gained popularity because of an attractive combination of expressive power and computational costs: There are practical unification algorithms [29–31] that compute most general unifiers whenever they exist. Pfenning [31] gave the first algorithm for higher-order pattern anti-unification in the Calculus of Constructions, with the intention of using it for proof generalization.

Since then, there have been several approaches to higher-order anti-unification, designing algorithms in various restricted cases. Motivated by applications in inductive learning, Feng and Muggleton [15] proposed anti-unification in $M\lambda$, which is essentially an extension of higher-order patterns by permitting free variables to apply to object terms, not only to bound variables. Object terms may contain constants, free variables, and variables which are bound outside of object terms. The algorithm has been implemented and was used for inductive generalization.

Pientka [32] studied anti-unification of linear higher-order patterns in the framework of developing substitution tree indexing for higher-order terms. Linear higher-order patterns require that every meta-variable occurs at most once in them, and they apply to all distinct bound variables in its context. The generalization algorithm has been used for the insertion of terms into the index.

Lu et al. [26] investigated anti-unification in a restricted version of $\lambda 2$ (a second-order $\lambda$-calculus with type variables [3]) and its applications in analogical programming and analogical theorem proving. The imposed restrictions guarantee uniqueness of the least general generalization. This algorithm as well as the one for higher-order patterns by Pfenning [31] have influenced the generalization algorithm used in the program transformation technique called supercompilation [27].

There are other fragments of higher-order anti-unification, motivated by analogical reasoning. A restricted version of second-order generalization has an application in the replay of program derivations [16]. A symbolic analogy model, called Heuristic-Driven Theory Projection, uses yet another restriction of higher-order anti-unification to detect analogies between different domains [21].

The last decade has seen a revived interest in anti-unification. The problem has been studied in various theories (e.g., [1, 10, 22]) and from different application points of view (e.g., [2, 9, 21, 25, 26, 35]). A particularly interesting application comes from software code refactoring, to find similar pieces of code, e.g., in Python, Java [7, 8] and Erlang [25] programs. These approaches are based on the first-order anti-unification [33, 34]. To advance the refactoring and clone detection techniques for languages based on $\lambda$ Prolog, one needs to employ anti-unification for higher-order terms. Yet another motivation to look into the problem of higher-order anti-unification in more detail would be the improvement of indexing techniques for $\lambda$-terms used, e.g., in mathematical assistant systems.

In this paper, we revisit the problem of higher-order anti-unification and present a rule-based anti-unification algorithm (in the style of Huet [19]) for simply typed $\lambda$-calculus. The input of the algorithm are arbitrary terms in $\eta$-long $\beta$-normal form. The output is a higher-order pattern. The global function for recording disagreements is represented as a store, in the spirit of Alpuente et al. [1]. We prove that a least general pattern generalization always exists and is *unique* modulo $\alpha$-equivalence. The proposed algorithm computes it in *linear* time. As it is done in related work, we assume that symbols and pointers are encoded in constant space, and basic operations on them are performed in constant time. With a small modification, the algorithm works for untyped lambda-calculus as well.

This paper is an extended and improved version of our conference publication [5]. There, it is proved that the problem is solvable in cubic time. A free open-source implementation for both simply typed and untyped calculi of this previous version of the algorithm is available.

### Comparison with Some Related Work

The approaches which are closest to us are the following:

- Pfenning [31] studied anti-unification in the Calculus of Constructions, whose type system is richer than the simple types we consider. Both the input and the output was required to be higher-order patterns. Some questions have remained open, including the efficiency, applicability, and implementations of the algorithm. Due to the nature of type dependencies in the calculus, the author was not able to formulate the algorithm in Huet’s style [19], where a global function is used to guarantee that the same disagreements between the input terms are mapped to the same variable. The complexity has not been studied and the proofs of the algorithm properties have been just sketched.
- Anti-unification in $M\lambda$ [15] is performed on simply typed terms, where both the input and the output are restricted to a certain extension of higher-order patterns. In this sense it is not comparable to our case, because we do not restrict the input, but require patterns in the output. Moreover, it contains neither the complexity analysis of the $M\lambda$ anti-unification algorithm nor the proofs of its properties.
- The anti-unification algorithm proposed by Pientka [32] also considers simply typed terms with the input and output restricted. The restriction requires terms to be linear higher-order patterns. Complexity results are not reported. This approach is also different from ours for the same reason as above: We do not restrict the input. It should be said that omitting one of the rules in our algorithm (the merging rule), we can also compute linear pattern generalizations for arbitrary input.

Some more remote results are listed below:

- Anti-unification for a restricted version of $\lambda 2$ [26] requires the $\lambda$-abstraction not to be used in arguments. The algorithm computes a generalization which is least general with respect to the combination of several orderings defined in the paper. The properties of the algorithm are formally proved, but the complexity has not been analyzed. As the authors point out, the orderings they define are not comparable with the ordering used to compute higher-order pattern generalizations.
- Generalization algorithms proposed by Hirata et al. [18] work on second-order terms which contain no $\lambda$-abstractions. The output is also restricted: It may contain variables which can be instantiated with multi-hole contexts only. Varying restrictions on the instantiation, various versions of generalizations are obtained. This approach is not comparable with ours.
- Yet another anti-unification algorithm for $\lambda$-abstraction-free terms has been developed for analogy making [21]. The application dictates the typical input to be first-order, while their generalizations may contain second-order variables. A certain measure is introduced to compare generalizations, and the algorithm computes those which are preferred by this measure. This approach is not comparable with ours either.
- The approach of Hasker [16] is also different from what we do. The anti-unification algorithm there works on a restriction of combinator terms and computes their generalizations (in quadratic time). It has been used for program derivation.

## Preliminaries

In higher-order signatures we have *types* constructed from a set of *basic types* (typically $\delta$) using the grammar $\tau \,{:}{:}{=}\, \delta \mid \tau \rightarrow \tau$ , where $\rightarrow$ is associative to the right. *Variables* (typically $X, Y, Z, x, y, z, a, b, \ldots$) and *constants* (typically $f, c, \ldots$) have an assigned type.

$\lambda$-*terms* (typically $t,s,u,\ldots$) are built using the grammar

$$\begin{aligned} t \,{:}{:}{=}\, x \mid c \mid \lambda x\cdot t \mid t_1\ t_2 \end{aligned}$$

where *x* is a variable and *c* is a constant, and are typed as usual. Terms of the form $(\ldots (h\ t_1 ) \ldots t_m )$, where *h* is a constant or a variable, will be written as $h(t_1 , \ldots , t_m )$, and terms of the form $\lambda x_1 . \cdots .\lambda x_n\cdot t$ as $\lambda x_1 , \ldots , x_n\cdot t$. We use $\vec {x}$ as a short-hand for $x_1 , \ldots , x_n$.

Other standard notions of the simply typed $\lambda$-calculus, like bound and free occurrences of variables, $\alpha$-conversion, $\beta$-reduction, $\eta$-long $\beta$-normal form, etc. are defined as usual (see [13]). By default, terms are assumed to be written in $\eta$-long $\beta$-normal form. Therefore, all terms have the form $\lambda x_1, \ldots , x_n .h(t_1 , \ldots , t_m )$, where $n, m \ge 0$, *h* is either a constant or a variable, $t_1 , \ldots , t_m$ have also this form, and the term $h(t_1 , \ldots , t_m )$ has a basic type.

The set of free variables of a term *t* is denoted by $\mathrm {Vars}(t)$. When we write an equality between two $\lambda$-terms, we mean that they are equivalent modulo $\alpha$, $\beta$ and $\eta$ equivalence.

The *size* of a term *t*, denoted ${|t|}$, is defined recursively as ${|h(t_1,\ldots ,t_n)|}= 1+ \sum _{i=1}^n{|t_i|}$ and ${|\lambda x\cdot t|} = 1 + {|t|}$.

The *depth* of a term *t*, denoted $\mathrm {Depth}(t)$ is defined recursively as $\mathrm {Depth}(h(t_1,\ldots ,t_n))= 1+ \max _{i\in \{1,\dots ,n\}} \mathrm {Depth}(t_i)$ and $\mathrm {Depth}(\lambda x\cdot t) = 1 + \mathrm {Depth}(t)$.

For a term $t= \lambda x_1,\ldots ,x_n. h(t_1,\ldots ,t_m)$ with $n,m\ge 0$, its *head* is defined as $\mathrm {Head}(t)=h$.

*Positions* in $\lambda$-terms are defined with respect to their tree representation in the usual way, as string of integers. For instance, in the term $f(\lambda x.\lambda y\cdot g(\lambda z.h(z,y),$ $x), \lambda u\cdot g(u))$, the symbol *f* stands in the position $\epsilon$ (the empty sequence), the occurrence of $\lambda x.$ stands in the position 1, the bound occurrence of *y* in 1.1.1.1.1.2, the bound occurrence of *u* in 2.1.1, etc.

The *path to a position* in a $\lambda$-term is defined as the sequence of symbols from the root to the node at that position (not including) in the tree representation of the term. For instance, the path to the position 1.1.1.1.1 in $f(\lambda x.\lambda y\cdot g(\lambda z\cdot h(z,y), x), \lambda u\cdot g(u))$ is $f,\lambda x,\lambda y,g,\lambda z$.

A *higher-order pattern* is a $\lambda$-term where, when written in $\eta$-long $\beta$-normal form, all free variable occurrences are applied to lists of pairwise distinct ($\eta$-long forms of) bound variables. For instance, $\lambda x. f (X (x), Y )$, $f (c, \lambda x.x)$ and $\lambda x.\lambda y\cdot X (\lambda z.x(z), y)$ are patterns, while $\lambda x\cdot f (X (X (x)), Y )$, *f* (*X* (*c*), *c*) and $\lambda x\cdot \lambda y\cdot X (x, x)$ are not.

*Substitutions* are finite sets of pairs $\{X_1\mapsto t_1,\ldots , X_n\mapsto t_n\}$ where $X_i$ and $t_i$ have the same type and the *X*’s are pairwise distinct variables. They can be extended to type preserving functions from terms to terms as usual, avoiding variable capture. The notions of substitution *domain* and *range* are also standard and are denoted, respectively, by $\mathrm {Dom}$ and $\mathrm {Ran}$.

We use postfix notation for substitution applications, writing $t\sigma$ instead of $\sigma (t)$. As usual, the application $t\sigma$ affects only the free occurrences of variables from $\mathrm {Dom}(\sigma )$ in *t*. We write $\vec {x}\sigma$ for $x_1\sigma ,\ldots ,x_n\sigma$, if $\vec {x}=x_1,\ldots ,x_n$. Similarly, for a set of terms *S*, we define $S\sigma = \{t\sigma \mid t\in S\}$. The *composition* of $\sigma$ and $\vartheta$ is written as juxtaposition $\sigma \vartheta$ and is defined as $x(\sigma \vartheta ) = (x\sigma )\vartheta$ for all *x*. Yet another standard operation, *restriction* of a substitution $\sigma$ to a set of variables *S*, is denoted by $\sigma |_S$.

A substitution $\sigma _1$ is *more general* than $\sigma _2$, written $\sigma _1\preceq \sigma _2$, if there exists $\vartheta$ such that $X\sigma _1\vartheta = X\sigma _2$ for all $X \in \mathrm {Dom}(\sigma _1)\cup \mathrm {Dom}(\sigma _2)$. The strict part of this relation is denoted by $\prec$. The relation $\preceq$ is a partial order and generates the equivalence relation which we denote by $\simeq$. We overload $\preceq$ by defining $s\preceq t$ if there exists a substitution $\sigma$ such that $s\sigma = t$.

A term *t* is called a *generalization* or an *anti-instance* of two terms $t_1$ and $t_2$ if $t\preceq t_1$ and $t\preceq t_2$. It is a *higher-order pattern generalization* if additionally *t* is a higher-order pattern. It is the *least general generalization,* (lgg in short), aka a *most specific anti-instance,* of $t_1$ and $t_2$, if there is no generalization *s* of $t_1$ and $t_2$ which satisfies $t\prec s$.

An *anti-unification problem* (shortly AUP) is a triple $X(\vec {x}): t \triangleq s$ where

- $\lambda \vec {x}\cdot X(\vec {x})$, $\lambda \vec {x}\cdot t$, and $\lambda \vec {x}\cdot s$ are terms of the same type,
- *t* and *s* are in $\eta$-long $\beta$-normal form, and
- *X* does not occur in *t* and *s*.

The variable *X* is called a *generalization variable.* The term $X(\vec {x})$ is called the *generalization term.* The variables that belong to $\vec {x}$, as well as bound variables, are written in the lower case letters $x,y,z,\ldots$. Originally free variables, including the generalization variables, are written with the capital letters $X,Y,Z,\ldots$. This notation intuitively corresponds to the usual convention about syntactically distinguishing bound and free variables. The size of a set of AUPs is defined as ${|\{X_1(\vec {x_1}):t_1 \triangleq s_1,\dots ,X_n(\vec {x_n}):t_n \triangleq s_n\}|} = \sum _{i=1}^n {|t_i|} + {|s_i|}$. Notice that the size of $X_i(\vec {x_i})$ is not considered.^1^

An *anti-unifier* of an AUP $X(\vec {x}) :t\triangleq s$ is a substitution $\sigma$ such that $\mathrm {Dom}(\sigma ) = \{X\}$ and $\lambda \vec {x}\cdot X(\vec {x})\sigma$ is a term which generalizes both $\lambda \vec {x}\cdot t$ and $\lambda \vec {x}\cdot s$.

An anti-unifier of $X(\vec {x}):t\triangleq s$ is *least general* (or *most specific*) if there is no anti-unifier $\vartheta$ of the same problem that satisfies $\sigma \prec \vartheta$. Obviously, if $\sigma$ is a least general anti-unifier of an AUP $X(\vec {x}):t\triangleq s$, then $\lambda \vec {x}\cdot X(\vec {x})\sigma$ is a lgg of $\lambda \vec {x}\cdot t$ and $\lambda \vec {x}\cdot s$.

Here we consider a variant of higher-order anti-unification problem:

Given: Higher-order terms *t* and *s* of the same type in $\eta$-long $\beta$-normal form.
Find: A higher-order pattern generalization *r* of *t* and *s*.

The problem statement means that we are looking for *r* which is least general among all higher-order patterns which generalize *t* and *s*. There can still exist a term which is less general than *r*, generalizes both *s* and *t*, but is not a higher-order pattern. For instance, if $t=\lambda x,y. f(h(x,x,y),h(x,y,y))$ and $s=\lambda x,y\cdot f(g(x,x,y),$ *g*(*x*, *y*, *y*)), then $r=\lambda x,y. f(Y_1(x,y),Y_2(x,y))$ is a higher-order pattern, which is an lgg of *t* and *s*. However, the term $\lambda x,y\cdot f(Z(x,x,y),Z(x,y,y))$, which is not a higher-order pattern, is less general than *r* and generalizes *t* and *s*.

Below we assume that in the AUPs of the form $X(\vec {x}) : t \triangleq s$, the term $\lambda \vec {x}\cdot X(\vec {x})$ is a higher-order pattern.

## Transformation Rules for a Variant of Higher-Order Anti-Unification

In this section we describe a set of transformation rules for higher-order anti-unification. These rules work on triples $A;S;\sigma$, which we call *states*. Here *A* is a set of AUPs of the form $\{X_1(\vec {x_1}) : t_1 \triangleq s_1, \ldots , X_n(\vec {x_n}) : t_n \triangleq s_n\}$ that are pending to anti-unify, *S* is a set of already solved AUPs (the *store*), and $\sigma$ is a substitution (computed so far) mapping variables to patterns.

### Remark 1

We assume that in the set $A\cup S$ each occurrence of $\lambda$ binds a distinct name variable (in other words, all names of bound variables are distinct), and that each $X_i$ occurs in $A\cup S$ only once.

### Definition 1

The set of transformations $\mathfrak {P}$ is defined by the following set of rules: 
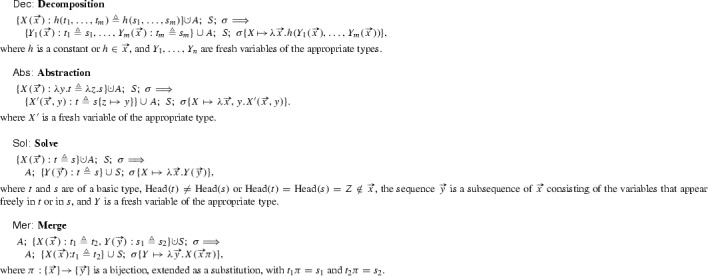


To compute generalizations for terms *t* and *s*, we start with the *initial state* $\{X : t \triangleq s\}; \varnothing ; \varnothing$, where *X* is a fresh variable, and apply the transformations as long as possible, until no transformation applies. These *final states* have the form $\varnothing ; S; \varphi$, where Mer does not apply to *S*. Then, the *result computed* by $\mathfrak {P}$ is $X\varphi$.

One can easily show that a triple obtained from $A;S;\sigma$ by applying any of the rules above to a state is indeed a state: For each expression $X(\vec {x}): t \triangleq s \in A\cup S$, the terms $X(\vec {x})$, *t* and *s* have the same type, $\lambda \vec {x}\cdot X(\vec {x})$ is a higher-order pattern, *s* and *t* are in $\eta$-long $\beta$-normal form, and *X* does not occur in *t* and *s*. Moreover, all generalization variables are distinct and substitutions map variables to patterns.

The property that each occurrence of $\lambda$ in $A\cup S$ binds a unique variable is also maintained. It guarantees that in the Abs rule, the variable *y* is fresh for *s*. After the application of the rule, *y* will appear nowhere else in $A\cup S$ except $X'(\vec {x},y)$ and, maybe, *t* and *s*.

Like in the anti-unification algorithms working on triple states [1, 22], the idea of the store here is to keep track of already solved AUPs in order to reuse an existing variable in generalizations. This is important, since we aim at computing lggs.

The Mer rule requires solving a matching problem  with the substitution $\pi$ which bijectively maps the variables from $\vec {x}$ to the variables from $\vec {y}$. In general, a matching problem is defined as follows.

### Definition 2

*(Permuting matcher)* Given a set of pairs of terms in $\eta$-long $\beta$-normal form  and two sets of variables *D* and *R* such that $D\subseteq \bigcup _{i=1}^n \mathrm {Vars}(t_i)$ and $R\subseteq \bigcup _{i=1}^n \mathrm {Vars}(s_i)$, a permuting matcher is a bijection $\pi :D\rightarrow R$ such that, extended as a substitution $\pi$ of variables $x\in D$ by variables $\pi (y)\in R$, satisfies $t_i\pi = s_i$, for $i=1,\dots ,n$.

The permuting matcher, if it exists, is unique^2^ and is denoted by ${\mathrm {match}}(D,R,P)$. When this map does not exist, we write ${\mathrm {match}}(D,R,P)=\bot$.

An algorithm that decides the existence of the permuting matcher and computes it in linear time is given in [5]. Here, in Sect. 5, we show that a more general problem can also be solved in linear time.

### Example 1

A couple of examples illustrating the generalizations computed by $\mathfrak {P}$:

- Let $$t= \lambda x,y. f(U(g(x),y), U(g(y),x))$$ and $$s=\lambda x',y'. f(h(y',g(x')),$$ $$ h(x',g(y')))$$. Then $$\mathfrak {P}$$ performs the following transformations:
  $$\begin{aligned} \begin{array}{ll} &{} \{X: \lambda x,y. f(U(g(x),y), U(g(y),x)) \triangleq \lambda x',y'. f(h(y',g(x')), h(x',g(y'))) \};\ \varnothing ;\ \varnothing \\ \Longrightarrow ^2_{\mathrm {Abs}}{} &{} \{X'(x,y): f(U(g(x),y), U(g(y),x)) \triangleq f(h(y,g(x)), h(x,g(y))) \};\ \varnothing ; \\ &{} \{X\mapsto \lambda x,y. X'(x,y)\} \\ \Longrightarrow _{\mathrm {Dec}}{} &{} \{Y_1(x,y): U(g(x),y) \triangleq h(y,g(x)), Y_2(x,y): U(g(y),x) \triangleq h(x,g(y)) \};\ \varnothing ;\\ &{} \{X\mapsto \lambda x,y. f(Y_1(x,y),Y_2(x,y)), X' \mapsto \lambda x,y. f(Y_1(x,y),Y_2(x,y))\} \\ \Longrightarrow _{\mathrm {Sol}}{} &{} \{Y_2(x,y): U(g(y),x) \triangleq h(x,g(y)) \}; \{Y_1(x,y): U(g(x),y) \triangleq h(y,g(x))\}; \\ &{} \{X\mapsto \lambda x,y. f(Y_1(x,y),Y_2(x,y)), X' \mapsto \lambda x,y. f(Y_1(x,y),Y_2(x,y))\} \\ \Longrightarrow _{\mathrm {Sol}}{} &{} \varnothing ;\ \{Y_1(x,y): U(g(x),y) \triangleq h(y,g(x)),Y_2(x,y): U(g(y),x) \triangleq h(x,g(y))\}; \\ &{} \{X\mapsto \lambda x,y. f(Y_1(x,y),Y_2(x,y)), X' \mapsto \lambda x,y. f(Y_1(x,y),Y_2(x,y))\} \\ \Longrightarrow _{\mathrm {Mer}} &{} \varnothing ;\ \{Y_1(x,y): U(g(x),y) \triangleq h(y,g(x))\};\ \{X\mapsto \lambda x,y. f(Y_1(x,y),Y_1(y,x)), \\ &{} \quad X' \mapsto \lambda x,y. f(Y_1(x,y),Y_1(y,x)), Y_2\mapsto \lambda x,y. Y_1(y,x)\} \end{array} \end{aligned}$$
  The computed result is $r= \lambda x,y. f(Y_1(x,y),Y_1(y,x))$. It generalizes the input terms *t* and *s*: $r\{Y_1\mapsto \lambda x,y. U(g(x),y)\}=t$ and $r\{Y_1 \mapsto \lambda x,y.h(y,g(x))\}=s$. These substitutions can be read from the final store.
- For $$\lambda x,y,z. g( f(x, z), f(y, z), f(y, x))$$ and $$\lambda x',y',z'. g( h(y',x'),$$ $$ h(x',y'), h(z',y'))$$, $$\mathfrak {P}$$ computes their generalization $$\lambda x,y,z. f(Y_1(x,y,z), Y_1(y,x,z), Y_1(y,z,x))$$
- For $\lambda x,y.f(\lambda z .U(z, y, x), U(x, y,x))$ and $\lambda x',y'.f(\lambda z' . h(y', z',x'),$ $h(y',x',x'))$, $\mathfrak {P}$ computes their generalization $\lambda x,y. f(\lambda z. Y_1(x,y,z),Y_2(x,y))$.

From the examples one can notice yet another advantage of using the store (besides helping in the merging): In the final state, it contains AUPs from which one can get the substitutions that show how the original terms can be obtained from the computed result.

## Properties of the Set of Transformations $\mathfrak {P}$

In this section we will prove termination (Theorem 1), soundness (Theorem 2) and completeness (Theorem 3) of $\mathfrak {P}$.

### Theorem 1

(Termination) The set of transformations $\mathfrak {P}$ is terminating.

Moreover, any transformation sequence starting in the state $A;S;\sigma$ terminates in $\mathscr {O}({|A|}+{|S|})$ steps.

### Proof

We define the measure of a state as $M(A;S;\sigma )=2\,{|A|}+{|S|}$. All rules in $\mathfrak {P}$ strictly decrease this measure. $\square$

### Theorem 2

(Soundness) If $\{X: t\triangleq s\};\varnothing ;\varnothing \Longrightarrow ^* \varnothing ; S;\sigma$ is a transformation sequence in $\mathfrak {P}$, then

- $X\sigma$ is a higher-order pattern in $\eta$-long $\beta$-normal form,
- $$X\sigma \preceq t$$ and $$X\sigma \preceq s$$.

### Proof

To prove that $X\sigma$ is a higher-order pattern, we use the facts that first, *X* is a higher order pattern and, second, at each step $A_1;S_1;\varphi \Longrightarrow A_2; S_2;\varphi \vartheta$ if $X\varphi$ is a higher-order pattern, then $X\varphi \vartheta$ is also a higher-order pattern. The latter property follows from stability of patterns under substitution application and the fact that substitutions in the rules map variables to higher-order patterns. As for $X\sigma$ being in $\eta$-long $\beta$-normal form, this is guaranteed by the series of applications of the Abs rule, even if Dec introduces an AUP whose generalization term is not in this form. It finishes the proof of (a).

Proving (b) is more involved. First, we prove that if $A_1;S_1;\varphi \Longrightarrow A_2; S_2;\varphi \vartheta$ is one step, then for any $X(\vec {x}): t\triangleq s \in A_1\cup S_1,$ we have $X(\vec {x})\vartheta \preceq t$ and $X(\vec {x})\vartheta \preceq s$. Note that if $X(\vec {x}): t\triangleq s$ was not transformed at this step, then this property trivially holds for it. Therefore, we assume that $X(\vec {x}): t\triangleq s$ is selected and prove the property for each rule:

- Dec: Here $t=h(t_1,\ldots ,t_m)$, $s=h(s_1,\ldots ,s_m)$, and $\vartheta = \{X \mapsto \lambda \vec {x}\cdot h(Y_1(\vec {x}),$ $\ldots ,Y_m(\vec {x}))\}$. Then $X(\vec {x})\vartheta = h(Y_1(\vec {x}),\ldots ,Y_m(\vec {x}))$. Let $\psi _1$ and $\psi _2$ be substitutions defined, respectively, by $Y_i\psi _1 = \lambda \vec {x}\cdot t_i$ and $Y_i\psi _2 = \lambda \vec {x}\cdot s_i$ for all $1\le i \le m$. Such substitutions obviously exist since the *Y*’s introduced by the Dec rule are fresh. Then $X(\vec {x})\vartheta \psi _1 = h(t_1,\ldots ,t_m)$, $X(\vec {x})\vartheta \psi _2 = h(s_1,\ldots ,s_m)$ and, hence, $X(\vec {x})\vartheta \preceq t$ and $X(\vec {x})\vartheta \preceq s$.
- Abs: Here $t= \lambda y_1.t'$, $s= \lambda y_2.s'$, and $\vartheta = \{X \mapsto \lambda \vec {x},y. X'(\vec {x},y)\}$. Then $X(\vec {x})\vartheta = \lambda y. X'(\vec {x},y)$. Let $\psi _1 = \{X'\mapsto \lambda \vec {x},y. t'\}$ and $\psi _2 = \{X'\mapsto \lambda \vec {x},y. s'\}$. Then $X(\vec {x})\vartheta \psi _1 = \lambda y.t' = t$, $X(\vec {x})\vartheta \psi _2 = \lambda y.s' = s$, and, hence, $X(\vec {x})\vartheta \preceq t$ and $X(\vec {x})\vartheta \preceq s$.
- Sol: We have $\vartheta = \{X\mapsto \lambda \vec {x}\cdot Y(\vec {y})\}$, where $\vec {y}$ is the subsequence of $\vec {x}$ consisting of the variables that appear freely in *t* or *s*. Let $\psi _1 = \{Y\mapsto \lambda \vec {y}\cdot t \}$ and $\psi _2 = \{Y\mapsto \lambda \vec {y}\cdot s \}$. Then $X(\vec {x})\vartheta \psi _1 = t$, $X(\vec {x})\vartheta \psi _2 = s$, and, hence, $X(\vec {x})\vartheta \preceq t$ and $X(\vec {x})\vartheta \preceq s$.

If Mer applies, then there exists $Y(\vec {y}):t'\triangleq s' \in S_1$ such that ${\mathrm {match}}(\{\vec {x}\},$ $\{\vec {y}\},$  is a permuting matcher $\pi$, and $\vartheta = \{Y\mapsto \lambda \vec {y}\cdot X(\vec {x}\pi )\}$. Then $X(\vec {x})\vartheta \preceq t$ and $X(\vec {x})\vartheta \preceq s$ obviously hold. As for the $Y(\vec {y}):t'\triangleq s'$, let $\psi _1 = \{X\mapsto \lambda \vec {x}\cdot t\}$ and $\psi _2 = \{X\mapsto \lambda \vec {x}\cdot s\}$. Then $Y(\vec {y})\vartheta \psi _1 = (\lambda \vec {x}\cdot t)(\vec {x}\pi ) = t\pi = t'$, $Y(\vec {y})\vartheta \psi _2 = (\lambda \vec {x}\cdot s)(\vec {x}\pi )=s\pi = s'$, and, hence, $Y(\vec {y})\vartheta \preceq t'$ and $Y(\vec {y})\vartheta \preceq s'$.

Now, we proceed by induction on the length *l* of the transformation sequence. In fact, we will prove a more general statement: If $A_0;S_0;\vartheta _0 \Longrightarrow ^* \varnothing ; S_n;\vartheta _0\vartheta _1\cdots \vartheta _n$ is a transformation sequence in $\mathfrak {P}$, then for any $X(\vec {x}):t \triangleq s\in A_0\cup S_0$ we have $X(\vec {x})\vartheta _1\cdots \vartheta _n \preceq t$ and $X(\vec {x})\vartheta _1\cdots \vartheta _n \preceq s$.

When $l=1$, it is exactly the one-step case we just proved. Assume that the statement is true for any transformation sequence of the length *n* and prove it for a transformation sequence $A_0;S_0;\vartheta _0 \Longrightarrow A_1;S_1;\vartheta _0\vartheta _1 \Longrightarrow ^* \varnothing ; S_n;\vartheta _0\vartheta _1\cdots \vartheta _n$ of the length $n+1$.

Below the composition $\vartheta _i\vartheta _{i+1}\cdots \vartheta _k$ is abbreviated as $\vartheta _i^k$ with $k\ge i$. Let $X(\vec {x}): t \triangleq s$ be an AUP selected for transformation at the current step. (Again, the property trivially holds for the AUPs which are not selected). We consider each rule:

- Dec: $t=h(t_1,\ldots ,t_m)$, $s=h(s_1,\ldots ,s_m)$ and $X(\vec {x})\vartheta _1^1 = h(Y_1(\vec {x}),\ldots ,$ $Y_m(\vec {x}))$. By the induction hypothesis, $Y_i(\vec {x})\vartheta _2^n\preceq t_i$ and $Y_i(\vec {x})\vartheta _2^n\preceq s_i$ for all $1\le i \le m$. By construction of $\vartheta _2^n$, if there is $U\in \mathrm {Vars}(\mathrm {Ran}(\vartheta _2^n))$, then there is an AUP of the form $U(\vec {u}):t'\triangleq s'\in S_n$. Let $\sigma$ (resp. $\varphi$) be a substitution which maps each such *U* to the corresponding $t'$ (resp. $s'$). Then $Y_i(\vec {x})\vartheta _2^n\sigma = t_i$ and $Y_i(\vec {x})\vartheta _2^n\varphi = s_i$. Since $X(\vec {x})\vartheta _1^n = h(Y_1(\vec {x}),\ldots ,Y_m(\vec {x}))\vartheta _2^n$, we get that $X(\vec {x})\vartheta _1^n \sigma = t$, $X(\vec {x})\vartheta _1^n \varphi = s$, and, hence, $X(\vec {x})\vartheta _1^n \preceq t$ and $X(\vec {x})\vartheta _1^n \preceq s$.
- Abs: Here $t= \lambda y_1.t'$, $s= \lambda y_2.s'$, $X(\vec {x})\vartheta _1^1= \lambda y. X'(\vec {x},y)$, and $A_1$ contains the AUP $X'(\vec {x},y): t'\{y_1\mapsto y\} \triangleq s'\{y_2\mapsto y\}$. By the induction hypothesis, $X'(\vec {x},y)\vartheta _2^n\preceq t'\{y_1\mapsto y\}$ and $X'(\vec {x},y)\vartheta _2^n\preceq s'\{y_1\mapsto y\}$. Since $X(\vec {x})\vartheta _1^n= \lambda y. X'(\vec {x},y)\vartheta _2^n$ and due to the way how *y* was chosen, we finally get $X(\vec {x})\vartheta _1^n\preceq \lambda y. t'\{y_1\mapsto y\}=t$ and $X(\vec {x})\vartheta _1^n\preceq \lambda y. s'\{y_2\mapsto y\}=s$.
- Sol: We have $X(\vec {x})\vartheta _1^1 = Y(\vec {y})$ where *Y* is in the store. By the induction hypothesis, $Y(\vec {y})\vartheta _2^n\preceq t$ and $Y(\vec {y})\vartheta _2^n\preceq s$. Therefore, $X(\vec {x})\vartheta _1^n \preceq t$ and $X(\vec {x})\vartheta _1^n \preceq s$.

For Mer, there exists $Y(\vec {y}):t'\triangleq s' \in S_0$ such that ${\mathrm {match}}(\{\vec {x}\},$  is a permuting matcher $\pi$, and $\vartheta _1^1 = \{Y\mapsto \lambda \vec {y}\cdot X(\vec {x}\pi )\}$. By the induction hypothesis, $X(\vec {x})\vartheta _1^n = X(\vec {x})\vartheta _2^n\preceq t$ and $X(\vec {x})\vartheta _1^n = X(\vec {x})\vartheta _2^n \preceq s$. These imply that $X(\vec {x}\pi )\vartheta _1^n \preceq t'$ and $X(\vec {x}\pi )\vartheta _1^n \preceq s'$, which, together $Y\vartheta _1^n = X(\vec {x}\pi )$, yields $Y(\vec {y})\vartheta _1^n \preceq t'$and $Y(\vec {y})\vartheta _1^n \preceq s'$.

$$\square $$

Hence, the result computed by $\mathfrak {P}$ for $X: t \triangleq s$ generalizes both *t* and *s*. We call $X\sigma$ a *generalization of* *t* *and* *s* *computed by* $\mathfrak {P}$. Moreover, given a transformation sequence $\{X: t \triangleq s \};\varnothing ;\varnothing \Longrightarrow ^* \varnothing ;S;\sigma$ in $\mathfrak {P}$, we say that

- $\sigma$ is a *substitution computed by* $\mathfrak {P}$ *for* $X: t \triangleq s$;
- the restriction of $\sigma$ on *X*, $\sigma |_X$, is an *anti-unifier of* $X:t \triangleq s$ *computed by* $\mathfrak {P}$.

### Theorem 3

(Completeness) Let $\lambda \vec {x}\cdot t_1$ and $\lambda \vec {x}\cdot t_2$ be higher-order terms and $\lambda \vec {x}\cdot s$ be a higher-order pattern such that $\lambda \vec {x}\cdot s$ is a generalization of both $\lambda \vec {x}\cdot t_1$ and $\lambda \vec {x}\cdot t_2$. Then, there exists a transformation sequence $\{X(\vec {x}): t_1\triangleq t_2\};\varnothing ;\varnothing \Longrightarrow ^* \varnothing ; S;\sigma$ in $\mathfrak {P}$ such that $\lambda \vec {x}\cdot s \preceq X\sigma$.

### Proof

By structural induction on *s*. We can assume without loss of generality that $\lambda \vec {x}\cdot s$ is an lgg of $\lambda \vec {x}\cdot t_1$ and $\lambda \vec {x}\cdot t_2$. We also assume that it is in the $\eta$-long $\beta$-normal form.

If *s* is a variable, then there are two cases: Either $s\in \vec {x}$, or $s\notin \vec {x}$. In the first case, we have $s=t_1 = t_2$. The Dec rule gives $\sigma = \{X\mapsto \lambda \vec {x}\cdot s\}$ and, hence, $\lambda \vec {x}\cdot s \preceq \lambda \vec {x}\cdot X(\vec {x})\sigma = s$. In the second case, either $\mathrm {Head}(t_1)\ne \mathrm {Head}(t_2)$, or $\mathrm {Head}(t_1)= \mathrm {Head}(t_2) \notin \vec {x}$. Sol is supposed to give us $\sigma =\{X\mapsto \lambda \vec {x}\cdot X'(\vec {x'})\}$, where $\vec {x'}$ is a subsequence of $\vec {x}$ consisting of variables occurring freely in $t_1$ or in $t_2$. But $\vec {x'}$ should be empty, because otherwise *s* would not be just a variable (remember that $\lambda \vec {x}\cdot s$ is an lgg of $\lambda \vec {x}\cdot t_1$ and $\lambda \vec {x}\cdot t_2$ in the $\eta$-long $\beta$-normal form). Hence, we have $\sigma =\{X\mapsto \lambda \vec {x}\cdot X'\}$ and $\lambda \vec {x}\cdot s\preceq \lambda \vec {x}\cdot X(\vec {x})\sigma$, because $s\{s\mapsto X'\} = X(\vec {x})\sigma$.

If *s* is a constant *c*, then $t_1=t_2 =c$. We can apply the Dec rule, obtaining $\sigma = \{X\mapsto \lambda \vec {x}\cdot c\}$ and, hence, $s = c \preceq X(\vec {x})\sigma = c$. Therefore, $\lambda \vec {x}\cdot s \preceq \lambda \vec {x}\cdot X(\vec {x})\sigma$.

If $s = \lambda x. s'$, then $t_1$ and $t_2$ must have the forms $t_1 = \lambda x. t'_1$ and $t_2=\lambda y. t'_2$, and $s'$ must be an lgg of $t'_1$ and $t'_2$. Abs gives a new state $\{X'(\vec {x},x):t'_1\triangleq t'_2\{x\mapsto y\}\};\varnothing ;\sigma _1$, where $\sigma _1 = \{X\mapsto \lambda \vec {x},x. X'(\vec {x},x)\}$. By the induction hypothesis, we can compute a substitution $\sigma _2$ such that $\lambda \vec {x},x.s'\preceq \lambda \vec {x},x.X'(\vec {x},x)\sigma _2$. Composing $\sigma _1$ and $\sigma _2$ into $\sigma$, we have $X(\vec {x})\sigma = \lambda x. X'(\vec {x},x)\sigma _2$. Hence, we get $\lambda \vec {x}\cdot s = \lambda \vec {x}\cdot \lambda x. s' \preceq \lambda \vec {x}\cdot \lambda x. X'(\vec {x},x)\sigma _2 = \lambda \vec {x}\cdot X(\vec {x})\sigma$.

Finally, assume that *s* is a compound term $h(s_1,\ldots ,s_n)$. If $h\notin \vec {x}$ is a variable, then $s_1,\ldots ,s_n$ are distinct variables from $\vec {x}$ (because $\lambda \vec {x}\cdot s$ is a higher-order pattern). That means that $s_1,\ldots ,s_n$ appear freely in $t_1$ or $t_2$. Moreover, either $\mathrm {Head}(t_1)\ne \mathrm {Head}(t_2)$, or $\mathrm {Head}(t_1)=\mathrm {Head}(t_2) = h$. In both cases, we can apply the Sol rule to obtain $\sigma = \{X\mapsto \lambda \vec {x}\cdot Y(s_1,\ldots ,s_n)\}$. Obviously, $\lambda \vec {x}\cdot s \preceq \lambda \vec {x}\cdot X(\vec {x})\sigma = \lambda \vec {x}\cdot Y(s_1,\ldots ,s_n)$.

If $h\in \vec {x}$ or if it is a constant, then we should have $\mathrm {Head}(t_1)=\mathrm {Head}(t_2)$. Assume they have the forms $t_1=h(t^1_1,\ldots ,t^1_n)$ and $t_2=h(t^2_1,\ldots ,t^2_n)$. We proceed by the Dec rule, obtaining $\{Y_i(\vec {x}):t^1_i \triangleq t^2_i \mid 1\le i \le n\};\varnothing ;\sigma _0$, where $\sigma _0 = \{X \mapsto \lambda \vec {x}\cdot h(Y_1(\vec {x}),\ldots , Y_n(\vec {x}))\}$. By the induction hypothesis, we can construct transformation sequences $\Delta _1,\ldots ,\Delta _n$ computing the substitutions $\sigma _1,\ldots ,\sigma _n$, respectively, such that $\lambda \vec {x}\cdot s_i\preceq \lambda \vec {x}\cdot Y_i(\vec {x})\sigma _i$ for $1\le i \le n$. These transformation sequences, together with the initial Dec step, can be combined into one transformation sequence, of the form $\Delta = \{X(\vec {x}):t_1 \triangleq t_2 \};\varnothing ;\sigma _0\Longrightarrow \{Y_i(\vec {x}):t^1_i \triangleq t^2_i \mid 1\le i \le n\};\varnothing ;\sigma _0 \Longrightarrow ^* \varnothing ; S_n; \sigma _0\sigma _1\cdots \sigma _n$.

Let for any term *t*, $t|_p$ denote the subterm of *t* at position *p*. If *s* does not contain duplicate variables free in $\lambda \vec {x}\cdot s$, then the construction of $\Delta$ and the fact that $\lambda \vec {x}\cdot s_i\preceq \lambda \vec {x}\cdot Y_i(\vec {x})\sigma _i$ for $1\le i \le n$ guarantee $\lambda \vec {x}\cdot s\preceq \lambda \vec {x}\cdot X(\vec {x})\sigma _0\sigma _1\cdots \sigma _n$. If *s* contains duplicate variables free in $\lambda \vec {x}\cdot s$ (e.g., of the form $\lambda \vec {u_1}\cdot Z(\vec {z_1})$ and $\lambda \vec {u_2}\cdot Z(\vec {z_2})$, where $\vec {z_1}$ and $\vec {z_2}$ have the same length) at positions $p_1$ and $p_2$, it indicates that

- $t_1|_{p_1}$ and $t_1|_{p_2}$ differ from each other by a permutation of variables bound in $t_1$,
- $t_2|_{p_1}$ and $t_2|_{p_2}$ differ from each other by the same (modulo variable renaming) permutation of variables bound in $t_2$,
- the path to $p_1$ is the same (modulo bound variable renaming) in $t_1$ and $t_2$. It equals (modulo bound variable renaming) the path to $p_1$ in *s*, and
- the path to $p_2$ is the same (modulo bound variable renaming) in $t_1$ and $t_2$. It equals (modulo bound variable renaming) the path to $p_2$ in *s*.

Then, because of (c) and (d), we should have two AUPs in $S_n$: One, between (renamed variants of) $t_1|_{p_1}$ and $t_2|_{p_1}$, and the other one between (renamed variants of) $t_1|_{p_2}$ and $t_2|_{p_2}$. The possible renaming of variables is caused by the fact that Abs might have been applied to obtain the AUPs. Let those AUPs be $Z(\vec {z_1}) : r^1_1 \triangleq r^2_1$ and $Z'(\vec {z_2}) : r^1_2 \triangleq r^2_2$. The conditions (a) and (b) make sure that  is a permuting matcher $\pi$, which means that we can apply the rule Mer with the substitution $\sigma '_1=\{Z'\mapsto \lambda \vec {z_2}\cdot Z(\vec {z_1}\pi )\}$. We can repeat this process for all duplicated variables in *s*, extending $\Delta$ to the transformation sequence $\Delta '= \{X(\vec {x}):t_1 \triangleq t_2 \};\varnothing ;\sigma _0\Longrightarrow \{Y_i(\vec {x}):t^1_i \triangleq t^2_i \mid 1\le i \le n\};\varnothing ;\sigma _0 \Longrightarrow ^* \varnothing ; S_n; \sigma _0\sigma _1\cdots \sigma _n \Longrightarrow ^* \varnothing ; S_{n+m}; \sigma _0\sigma _1\cdots \sigma _n\sigma '_1\cdots \sigma '_m$, where $\sigma '_1,\ldots , \sigma '_m$ are substitutions introduced by the applications of the Mer rule. Let $\sigma =\sigma _0\sigma _1\cdots \sigma _n\sigma '_1\cdots \sigma '_m$. By this construction, we have $\lambda \vec {x}\cdot s\preceq \lambda \vec {x}\cdot X(\vec {x})\sigma$, which finishes the proof. $\square$

Depending which AUP is selected to perform a transformation, there can be different transformation sequences in $\mathfrak {P}$ starting from the same initial state, but leading to different generalizations. The next theorem states that all those generalizations are equivalent.

### Theorem 4

(Uniqueness Modulo $\simeq$ **)** Let $\{X: t\triangleq s\};\varnothing ;\varnothing \Longrightarrow ^* \varnothing ; S_1;\sigma _1$ and $\{X: t\triangleq s\};$ $\varnothing ;\varnothing \Longrightarrow ^* \varnothing ; S_2;\sigma _2$ be two transformation sequences in $\mathfrak {P}$, where $\varnothing ; S_1;\sigma _1$ and $\varnothing ; S_2;\sigma _2$ are final states. Then $X\sigma _1 \simeq X\sigma _2$.

### Proof

It is not hard to notice that if it is possible to change the order of applications of rules (but sticking to the same selected AUPs for each rule) then the result remains the same: If $\Delta _1 = A_1;S_1;\sigma _1 \Longrightarrow _{\mathrm {R1}} A_2;S_2;\sigma _1\vartheta _1 \Longrightarrow _{\mathrm {R2}} A_3; S_3; \sigma _1\vartheta _1\vartheta _2$ and $\Delta _2= A_1;S_1;\sigma _1 \Longrightarrow _{\mathrm {R2}} A'_2;S'_2;\sigma _1\vartheta _2 \Longrightarrow _{\mathrm {R1}} A'_3; S'_3; \sigma _1\vartheta _2\vartheta _1$ are two two-step transformation sequences, where R1 and R2 are (not necessarily different) rules and each of them transforms the same AUP(s) in both $\Delta _1$ and $\Delta _2$, then $A_3=A'_3$, $S_3=S'_3$, and $\sigma _1\vartheta _1\vartheta _2=\sigma _1\vartheta _2\vartheta _1$ (modulo the names of fresh variables).

Dec, Abs and Sol rules transform the selected AUP in a unique way. We show that it is irrelevant in which order we perform matching in the Mer rule.

Let  $\sigma \{Y \mapsto \lambda \vec {y}\cdot Z(\vec {z}\pi )\}$ be the merging step with $\pi ={\mathrm {match}}(\{\vec {z}\}, \{\vec {y}\},$ . If we do it in the other way around, we would get the step , where $\mu ={\mathrm {match}}(\{\vec {y}\},$ . But $\mu =\pi ^{-1}$, because of bijection.

Let $\vartheta _1 = \sigma \rho _1$ with $\rho _1=\{Y \mapsto \lambda \vec {y}\cdot Z(\vec {z}\pi )\}$ and $\vartheta _2=\sigma \rho _2$ with $\rho _2=\{Z \mapsto \lambda \vec {z}\cdot Y(\vec {y}\pi ^{-1} )\}$. Our goal is to prove that $X\vartheta _1 \simeq X\vartheta _2$. For this, we have to prove two inequalities: $X\vartheta _1 \preceq X\vartheta _2$ and $X\vartheta _2 \preceq X\vartheta _1$. To show $X\vartheta _1 \preceq X\vartheta _2$, we first need to prove the equality:

1 $$\begin{aligned}&\lambda \vec {y}\cdot Z (\vec {z}\pi )\rho _2 = \lambda \vec {y}\cdot Y(\vec {y}). \end{aligned}$$

Its left hand side is transformed as $\lambda \vec {y}\cdot Z (\vec {z}\pi )\rho _2 = \lambda \vec {y}\cdot Z (\vec {z}\pi )\{Z\mapsto \lambda \vec {z}\cdot Y(\vec {y}\pi ^{-1} )\} = \lambda \vec {y}\cdot (\lambda \vec {z}\cdot Y(\vec {y}\pi ^{-1} ) (\vec {z}\pi ))$. The $\beta$-reduction of $\lambda \vec {z}\cdot Y(\vec {y}\pi ^{-1} )(\vec {z}\pi )$ replaces each occurrence of $z_i\in \vec {z}$ in $Y(\vec {y}\pi ^{-1} )$ with $z_i\pi$, which is the same as applying $\pi$ to $Y(\vec {y}\pi ^{-1} )$. Since $\vec {y}\pi ^{-1} \pi =\vec {y}$, we get $\lambda \vec {y}\cdot (\lambda \vec {z}\cdot Y(\vec {y}\pi ^{-1} ) (\vec {z}\pi )) = \lambda \vec {y}\cdot Y(\vec {y}\pi ^{-1} \pi ) = \lambda \vec {y}\cdot Y(\vec {y})$ and (1) is proved.

Next, starting from $X\vartheta _1\rho _2$, we can transform it as $X\vartheta _1\rho _2= X\sigma \rho _1\rho _2 = X\sigma \{Y \mapsto \lambda \vec {y}\cdot Z(\vec {z}\pi )\rho _2,Z \mapsto \lambda \vec {z}\cdot Y(\vec {y}\pi ^{-1} )\} =_{\text {by (1)}} X\sigma \{Y \mapsto \lambda \vec {y}\cdot Z(\vec {z}\pi )\rho _2,Z \mapsto \lambda \vec {z}\cdot Y(\vec {y}\pi ^{-1} )\}= X\sigma \{Y \mapsto \lambda \vec {y}\cdot Y(\vec {y}),Z \mapsto \lambda \vec {z}\cdot Y(\vec {y}\pi ^{-1} )\} = X\sigma \{Y \mapsto \lambda \vec {y}\cdot Y(\vec {y})\}\{Z \mapsto \lambda \vec {z}\cdot Y(\vec {y}\pi ^{-1} )\}.$ At this step, since the equality $=$ is $\alpha \beta \eta$-equivalence, we can omit the application of the substitution $\{Y \mapsto \lambda \vec {y}\cdot Y(\vec {y})\}$ and proceed: $X\sigma \{Y \mapsto \lambda \vec {y}\cdot Y(\vec {y})\}\{Z \mapsto \lambda \vec {z}\cdot Y(\vec {y}\pi ^{-1} )\} = X\sigma \{Z \mapsto \lambda \vec {z}\cdot Y(\vec {y}\pi ^{-1} )\} = X\sigma \rho _2 X\vartheta _2.$ Hence, we got $X\vartheta _1\rho _2=X\vartheta _2$, which implies $X\vartheta _1 \preceq X\vartheta _2$.

The fact $X\vartheta _2 \preceq X\vartheta _1$ can be proved analogously. Hence, $X\vartheta _1\simeq X\vartheta _2$, which means that it is irrelevant in which order we perform matching in the Merge rule. Therefore, no matter how different transformation sequences are constructed, the computed generalizations are equivalent. $\square$

### Corollary 1

For any given terms *t* and *s*, and any transformation sequence $\{X: t\triangleq s\};\varnothing ;\varnothing \Longrightarrow ^* \varnothing ; S;\sigma$ in $\mathfrak {P}$, the higher-order pattern $X\sigma$ is the unique least general generalization of *t* and *s*.

## Complexity

In this section we describe an algorithm based on the set of transformations $\mathfrak {P}$ and prove that this algorithm has linear time complexity. Notice that the Termination Theorem (Theorem 1) already proves that any transformation sequence in $\mathfrak {P}$ has at most linear length. However, the direct application of one transformation rule, as described in Sect. 3, may require quadratic time, what would result in a cubic time algorithm, similar to our previous one [5]. In this section we will improve this result.

### Remark 2

In the complexity analysis that follows we will assume the following:

1. All pointers require constant space and all basic operations on them can be done in constant time. This assumption is popular in the literature, despite the fact that it is inaccurate: In any implementation of trees based on the use of pointers, these will need space $\mathscr {O}(\log n)$, since they address a memory of size $\mathscr {O}(n)$. The same argument applies to all traditional algorithms for first-order unification. In fact, all those claimed to be linear, without this assumption would have $\mathscr {O}(n\,\log n)$ time complexity. Therefore, we will continue with this traditional assumption.
2. All symbols can be represented in constant space and all basic operations on them done in constant time. Again, this assumption is traditional, but inaccurate. A term with $\mathscr {O}(n)$ symbols, where all of them can be distinct, would require $\mathscr {O}(\log n)$ space to represent each single symbol.
3. When we represent lambda-terms in de Bruijn form, indexes of bound variables can be represented in constant space.

In other words, we will neglect logarithmic factors in front of polynomial functions.

The proposed algorithm works in three phases. Later, we will prove that each one of them can be done in linear time. The following lemma allows us to decompose any transformation sequence in the three phases that we will analyze separately.

### Lemma 1

(Phase division) Any sequence of transformations $\{X: t \triangleq s \};\varnothing ;\varnothing \Longrightarrow ^* \varnothing ;S;\sigma$ in $\mathfrak {P}$ can be decomposed into an equivalent sequence of transformations of the form

$$\begin{aligned} \{X: t \triangleq s \};\varnothing ;\varnothing \Longrightarrow ^*_{\textsf {Dec,Abs}} P_k;\varnothing ;\sigma _k \Longrightarrow ^*_{\textsf {Sol}} \varnothing ;S_l;\sigma _l \Longrightarrow ^*_{\textsf {Mer}} \varnothing ;S;\sigma . \end{aligned}$$

### Proof

We apply rules Dec and Abs exhaustively until all equations can only be transformed by the Sol rule. Notice that conditions of applicability of Dec and Abs and of Sol are disjoint. Then, in the second phase we only apply the Sol rule. Notice that this rule does not introduce new equations where Dec and Abs could be applicable. Then, in the third phase, when the first component of the tuple is empty, we only apply the Mer rule. Again, notice that this rule does not introduce new equations in the first component. By Theorem 4, following this strategy, we get an equivalent anti-unifier. $\square$

### Example 2

From $X:\lambda x_1\dots x_n.f(t_1,\dots ,t_n) \triangleq \lambda y_1\dots y_n.f(s_1,\dots ,s_n)$ after *n* applications of Abs and one of Dec, we get the problem set

$$\begin{aligned} \begin{array}{c} \{Y_1(x_1,\dots ,x_n):t_1 \triangleq s_1\{y_1\mapsto x_1\}\dots \{y_n\mapsto x_n\},\\ \dots ,\\ Y_n(x_1,\dots ,x_n):t_n \triangleq s_n\{y_1\mapsto x_1\}\dots \{y_n\mapsto x_n\}\}. \end{array} \end{aligned}$$

Notice that, if we counted the number of symbols of $Y_i(x_1,\dots ,x_n)$ to compute the size of the new AUPs, this would be quadratic on the size of the original AUP. This is the reason to only consider the number of symbols of the second and third component of the AUP to define its size. Moreover, reusing the representation of $x_1,\dots ,x_n$ in a directed acyclic graph, we can also represent the new AUPs in linear space on the size of the representation of the original AUP.

### Lemma 2

(First phase) There exists an algorithm that, given a representation of the AUP $\{X:t \triangleq s \}$, computes a representation of *P* and $\sigma$, where

$$\begin{aligned} \{X: t \triangleq s \};\varnothing ;\varnothing \Longrightarrow ^*_{\textsf {Dec,Abs}} P;\varnothing ;\sigma \end{aligned}$$

in time $\mathscr {O}(|t|+|s|)$. Moreover, ${|P|} \le {|X\!:\!t \! \triangleq \! s|}= |t|+|s|$.

### Proof

In the Dec rule, we reuse the representation of $t_i$ from $f(t_1,\dots ,t_m)$ to get a representation of each $t_i$. Using a directed acyclic graph, we also reuse the argument vector $\vec {x}$ from the representation of $X(\vec {x})$ in the original AUP and $\sigma$ to construct the representation of each $Y_i(\vec {x})$ in the new AUPs and substitution. We assume that in constant time, by using an appropriate hash table we can find the unique occurrence of *X* in $\sigma$, and hence compute $\sigma \{X\mapsto \lambda \vec {x}\cdot h(Y_1(\vec {x}),\ldots ,Y_m(\vec {x}))\}$ in time $\mathscr {O}(m)$. Notice that $\beta$-reduction is trivial in this use case. Therefore, the rule can be applied in time $\mathscr {O}(m)$, and the space requirements also increase in $\mathscr {O}(m)$.

In the Abs rule, we reuse the representation of $\lambda y.t$ to construct the representation of *t*. We also reuse the representation of $\vec {x}$ from $X(\vec {x})$ when we construct the representation of $X'(\vec {x},y)$. The most expensive step is to compute the substitution $s\{z\mapsto y\}$. We assume that, using an appropriated data structure where all occurrences of the bound variable *z* are linked, this can be done in linear time on the number of occurrence of this variable. This structure can be constructed for the initial problem in linear time.

If we bound the complexity as the product of the number of times we can apply these rules (linear) by the cost of every application (also linear in the worst case), we get a quadratic bound. In order to refine this bound we need to introduce the notion of *extended* size of a term, noted ${\Vert t\Vert }$, defined inductively as:

$$\begin{aligned} \begin{array}{l} {\Vert h(t_1,\ldots ,t_m)\Vert } = m + {\Vert t_1\Vert }+\dots +{\Vert t_m\Vert } + 1,\\ {\Vert \lambda y.t\Vert } = r + {\Vert t\Vert } + 1 \ \ \ \text{ where } r \text{ is } \text{ the } \text{ number } \text{ of } \text{ free } \text{ occurrences } \text{ of } y \text{ in } t. \end{array} \end{aligned}$$

It can be easily proved that ${\Vert t\Vert } \le 3\,{|t|}$. We can prove that the applications of Dec and Abs rules decrease the sum of the extended sizes of the terms of the equations in the same amount as the time they needed to be applied. All these together prove that this phase of the algorithm can be computed in linear time, and that the increase of space is also linear.

Finally, ${|P|} \le |t|+|s|$ is proved by inspection of the rules. $\square$

### Lemma 3

**(Second phase)** There exists an algorithm that, given a representation of $P;\varnothing ;\sigma$, computes a representation of *S* and $\sigma '$, where

$$\begin{aligned} P;\varnothing ;\sigma \Longrightarrow ^*_{\textsf {Sol}} \varnothing ;S;\sigma ' \end{aligned}$$

in time $\mathscr {O}(|P|)$. Moreover, ${|S|} = {|P|}$.

### Proof

In this second phase, we basically move equations from the problem set *P* to the store *S*. However, notice that the arguments of generalization variables in $X(\vec {x}):t\triangleq s$ are narrowed in $Y(\vec {y}):t\triangleq s$, where $\vec {y}$ is a subset of $\vec {x}$. As only those argument variables which appear in one of the terms *t* and *s* are kept, the length of the narrowed vector $\vec {y}$ is bound by ${|t|}+{|s|}$.

There is no need to share the representation of those narrowed argument vectors $\vec {y}$ anymore. The representation of $\vec {y}$ can be constructed without reusing the representation of $\vec {x}$ in linear time on the size of the AUP $X(\vec {x}) : t \triangleq s$. The substitution composition $\sigma \{X\mapsto \lambda \vec {x}\cdot Y(\vec {y})\}$ used by Sol is equivalent to replacing the subterm $X(\vec {x})$ by $Y(\vec {y})$. Again, this composition can also be done in linear time on ${|X(\vec {x}) : t \triangleq s|}$. Therefore, all the phase can be computed on time $\mathscr {O}(|P|)$.

Finally, ${|S|}={|P|}$ by inspection of the rule. $\square$

As a direct consequence of the previous two lemmas, we can conclude that the size of the store *S*, after the first and second phase of the anti-unification algorithm, is linear on the size of the original problem.

In order to prove that the third phase can also be implemented with a linear time algorithm, we can not directly use the rules described in previous section. This would lead to a cubic time algorithm, similar to our previous one [5].

First, we will reduce computation of permuting matchers to $\alpha$-equivalence, using the following lemma.

### Lemma 4

Given two terms *t* and *s* in $\eta$-long $\beta$-normal form, and two sets of variables $D\subseteq \mathrm {Vars}(t)$ and $R\subseteq \mathrm {Vars}(s)$, we have  if, and only if, $\lambda \vec {x}\cdot t$ is $\alpha$-equivalent to $\lambda \vec {y}\cdot s$, where $\vec {x}$ (resp. $\vec {y}$) is an ordering of the set *D* (resp. *R*) such that, for any $i<j$, the variable $x_i$ occurs free for the first time in *t* (resp *s*) before the first occurrence of $x_j$ with respect to the depth-first pre-order traversal of *t* (resp *s*).

Moreover, we can decide if  in time $\mathscr {O}({|t|}+{|s|})$.

### Proof

Two terms $\lambda \vec {x}\cdot t$ and $\lambda \vec {y}\cdot s$ are $\alpha$-equivalent if, and only if, $t[\vec {x}\mapsto \vec {y}]$ and *s* are $\alpha$-equivalent. If all variables $\{\vec {x}\}$ occur free in *t*, all variables $\{\vec {y}\}$ free in *s*, and *t* and *s* are in $\beta$-normal form, then $t[\vec {x}\mapsto \vec {y}]$ and *s* are $\alpha$-equivalent if, and only if, . Now, notice that, if , then the permuting matcher must be $[\vec {x}\mapsto \vec {y}]$, with sequences $\vec {x}$ and $\vec {y}$ constructed as stated in the lemma. Altogether, this proves the first part of the lemma.

The second part of the lemma relies on the first part. Given a term *t* (resp. *s*), we can close it, adding lambda-bindings for all free variables in *D* (resp. *R*) in the same order they appear for the first time in the term w.r.t. the depth-first pre-order traversal. Then, we can transform it into de Bruijn form. Both processes can be done in linear time.^3^ Then, to check if the two closing sequences (sequences of lambda-bindings) yield a permuting matcher for the two terms, we only need to check, in linear time, if the de Bruijn forms of the closed representations are equal. $\square$

However, we still have the problem that this should be repeated for any pair of AUPs in the store. A naive implementation of this process would result into a quadratic algorithm in the size of the store. However, this can be done in quasi-linear time using the following result.

### Lemma 5

Given a sequence of numbers $S=n_1,\dots ,n_m$ written in binary, we can partition this set into subsets of equal numbers in time $\mathscr {O}(|S|\,\log |S|)$.

### Proof

If we assume that numbers are bounded and representable in constant space, then we can use a perfect hash table. We add each number in the hash table, and construct the subsets from them in time $\mathscr {O}(m)=\mathscr {O}(|S|)$.

If numbers are unbounded, then we can use a similar idea, without using hash functions. We use a trie, i.e. a binary tree *T* such that, for any $i=1,\dots ,m$, tree *T* contains a node at position $n_i$ with label *i*. Starting with the empty tree, we proceed adding all necessary nodes to ensure that $n_i$ is a position in the tree. This can be done in linear time on the representation of $n_i$, i.e. $\mathscr {O}(\log n_i)=\mathscr {O}(|n_i|)$. Then, we add label *i* to the corresponding node, on time linear on the representation of *i*. At the end, sets of labels will represent subsets of equal numbers in *S*.

Since $m=\mathscr {O}(n)$,we have $|i|= \mathscr {O}(\log |S|)$. Therefore, this algorithm requires time $\mathscr {O}(|S|\,\log |S|)$. $\square$

Using the same ideas as Hinze [17], the previous lemma can be generalized when, instead of numbers written in binary, we have other objects represented in a fixed signature. In our case, we will use the algorithm described in the proof of Lemma 5 to find subsets of $\alpha$-equivalent $\lambda$-terms. For this purpose, we translate $\alpha$-terms into their de Bruijn form, and then represent them by their pre-order traversal sequence.

We are now able to establish the complexity of the third phase of the algorithm.

### Lemma 6

(Third phase) There exists an algorithm that, given a state $\varnothing ;S;\sigma$, computes $S'$ and $\sigma '$, where $\varnothing ;S;\sigma \Longrightarrow ^*_{\textsf {Mer}} \varnothing ;S';\sigma '$, in time $\mathscr {O}(|S|)$.

### Proof

As we have described in the proof of Lemma 4, we close all terms, adding lambda binders for all free occurrences of variables, and translate them into de Bruijn form. This can be done in linear time. By Lemma 4, to decide the existence of a permuting matcher for two terms, we only need to check $\alpha$-equivalence, i.e., equality once the term is represented in de Bruijn form. Here we assume that every de Bruijn index only requires constant space in order to be represented. Assuming also that the rest of symbols also require constant space, we can represent the terms by their pre-order traversal sequence. This traversal sequence has size linear on the size of the term. By Lemma 5, we can partition the store *S* into subsets of equal terms, i.e. subsets of permuting matchers. Then, applying rule Mer to all AUPs of the subset, we remove all but one representative of each subset. In each application, the cost of applying the rule is linear on the size of the removed equation, since the side condition of the rule has already been checked. $\square$

From Lemmas 1, 2, 3 and 6 we can conclude the following result.

### Theorem 5

(Complexity of $\mathfrak {P}$ **)** Computing the least general higher-order pattern generalization of two simply typed lambda-terms in $\eta$-long $\beta$-normal form has linear time complexity on the size of the input.

### Remark 3

The previous result is only valid under the assumption that all pointers, de Bruijn indexes, and representation of symbols only require constant space. Without these assumptions, we could prove that our anti-unification algorithm—like all traditional unification algorithms—is, in fact, quasi-linear. One could argue that in traditional unification algorithms, input terms are also represented as trees encoded with pointers; hence, input size is also quasi-linear on the number of symbols of the terms, like the complexity of the algorithm; therefore, the algorithm is quasi-linear in the number of symbols, but linear in the size of the input. However, this is not true. As Jacobsen proves, for trees with a constant number of distinct nodes, there exist succinct representations that only require linear space on the number of nodes [20]. In this representation, even to simply access a node’s child requires logarithmic time. Since in complexity theory inputs are assumed to be represented succinctly, even the traditional algorithm for tree traversal strictly requires quasi-linear time.

## Conclusion and Final Remarks

We designed an algorithm for computing higher-order pattern generalizations for simply typed lambda terms. The algorithm does not assume any restriction on the input except requiring them to be terms of the same type in the $\eta$-long $\beta$-normal form. The computed pattern is a least general generalization of the input terms, and is unique modulo free variable renaming and $\alpha$-equivalence. It is computed in linear time in the size of input (under the usual conventions made in the unification literature on the space requirements for encoding symbols and pointers, and on the complexity of basic operations on them).

One can observe that the set of transformations $\mathfrak {P}$ used in the paper can be adapted with a relatively little effort to work on untyped terms (cf. the formulation of the unification algorithm both for untyped and simply typed patterns [30]). One thing to be added is lazy $\eta$-expansion: The AUP of the form $X(\vec {x}): \lambda y.t \triangleq h(s_1,\ldots ,s_m)$ should be transformed into $X(\vec {x}): \lambda y.t \triangleq \lambda z.h(s_1,\ldots ,s_m,z)$ for a fresh *z*. (Dually for abstractions in the right hand side). In addition, Sol needs an extra condition for the case when $\mathrm {Head}(t)=\mathrm {Head}(s)$ but the terms have different number of arguments such as, e.g., in *f*(*a*, *x*) and *f*(*b*, *x*, *y*). Note that the complexity of the algorithm will remain linear in the untyped case, since the side enlargements in the lazy $\eta$-expansion are bounded by the size of the original problem in such a way that in the worst case, by summing up all enlargements one would only duplicate the size.

The anti-unification algorithm has been implemented (both for simply typed and untyped terms, without perfect hashing, using a simpler but more expensive method to compute permuting matchers) in Java as a part of an open-source anti-unification library [4]. It can be used online or can be downloaded freely from http://www.risc.jku.at/projects/stout/software/hoau.php.

As for the related topics, we mention nominal anti-unification. Several authors explored relationship between nominal terms and higher-order patterns (see, e.g., [12, 14, 23, 24] among others), proposing translations between them in the context of unification. However, it is not immediately clear how to reuse those translations for anti-unification, in particular, how to get nominal generalizations from pattern generalizations. Therefore, we proposed a direct algorithm for nominal anti-unification [6].

Studying anti-unification in the calculi with more complex type systems, such as the extension of the system *F* with subtyping $F_{<:}$ [11], would be a very interesting direction of future work, as it may have applications in clone detection and refactoring for the functional programming languages in the ML family.

## Footnotes

1 The proof of Theorem 1 and Example 2 motivate this decision. When in an AUP $X(\vec {y}):t \triangleq s$ all variables in $\vec {y}$ occur free in *t* or in *s*, this decision is not relevant. However, notice that at certain points we deal with AUPs that do not have this property.

2 In the uniqueness of permuting matchers it plays an important role the fact that *D* and *R* are subsets of the free variables occurring in $t_i$ and $s_i$, respectively, and that these terms are in $\beta$-normal form.

3 Strictly speaking, this is false in the worst case, since we can have a linear number of bound variables, and it would require de Bruijn indexes that can only be represented in logarithmic space. Then, the resulting algorithm would be quasi-linear. However, as we do for encodings of symbols and pointers, we will follow the standard practice and assume that de Bruijn indexes only require constant space.
